# Bacteria in COPD; their potential role and treatment

**DOI:** 10.1186/2213-0802-1-13

**Published:** 2013-08-30

**Authors:** Paul T King, Martin MacDonald, Philip G Bardin

**Affiliations:** 1 Monash Lung and Sleep, Monash Medical Centre, 246 Clayton Road, Clayton, Melbourne, 3168 Australia; 2 Department of Medicine, Monash Medical Centre, Monash University, Melbourne, Australia; 3 Monash Institute of Medical Research, Monash Medical Centre, Melbourne, Australia

**Keywords:** Chronic Obstructive Pulmonary Disease, Chronic Obstructive Pulmonary Disease Patient, Azithromycin, Macrolides, Lower Respiratory Tract

## Abstract

The role of bacterial infection in chronic obstructive pulmonary disease (COPD) and how it should be treated has been an ongoing source of controversy. For many years bacterial infection has not been thought to have an important effect in the pathology of this condition. Recent advances in diagnostic techniques, particularly the use 16S sequencing has demonstrated that there are a large range of bacteria present in the lower respiratory tract, both in terms of exacerbations and chronic colonization. A proportion of the bacteria present in the lower respiratory have also been shown to produce inflammation and hence are likely to be relevant for the pathogenesis of COPD. The accurate diagnosis of bacterial infection in individual patients remains a major challenge. The trials that have assessed the effect of antibiotics in COPD have generally been of low quality and have not been placebo controlled. Recent studies of macrolides for long-term treatment in COPD have found significantly reduced rates of exacerbations. Major challenges remain in accurately defining the potential role of bacteria in the inflammatory process and how best to optimize the use of antibiotics without the overuse of this limited resource. Alternative strategies to treat infection in COPD remain very limited.

## Review

The role of bacterial infection in COPD and how it should be treated has been an ongoing source of controversy. The British hypothesis proposed that recurrent bronchial infections were the reason that some smokers developed airflow obstruction while others did not [1, 2]. The subsequent study of Fletcher and Peto demonstrated that chronic bronchitis and respiratory infections did not relate to lung function decline [3]. As a consequence for many years it was believed that the chronic bronchitis syndrome had no relevance to lung function decline in COPD. However more recent studies have described a relationship between lung function decline and respiratory infections. Chronic bronchitis [4], respiratory infections [5, 6] and sputum bacterial counts [7]; have been shown to be related to decline in lung function in COPD. The role of chronic bronchitis remains controversial though.

A relatively recently realized feature of COPD is intense bronchial inflammation; that persists despite the cessation of smoking and is most prominent in advanced disease [8, 9]. The factors that drive the inflammatory process after smoking cessation have not been clearly defined [10]. Bacterial infection is one potential candidate and bacteria can induce inflammation both in the context of exacerbations and in the stable baseline state.

Antibiotics have been used as standard management for the treatment of exacerbations of COPD, but their value in this context remains uncertain [11]. Recent trials of the long-term use of macrolides have shown promising results [12].

This review will discuss the role of bacteria in the pathogenesis of COPD. Methods of diagnosing respiratory infection will be reviewed and the potential effect of antibiotics and other therapies in treatment.

### Microbiology of the respiratory tract

Bacteria have established niches in the human body where they exist in symbiosis with their host. The term microbiome is used to refer to complex communities of microorganisms, termed the microbiota, that inhabit the body surfaces in symbiosis with the host [13]. The most clearly defined microbiome occurs in the gastrointestinal tract in which there are far more bacterial cells than in the whole human body (as bacteria are much smaller than human cells). Bacteria which form part of the normal flora of the microbiome, are tolerated by the local mucosal immune response with minimal activation of inflammatory cells, in particular T cells [14]. Significant recent advances in the understanding of the human microbiome have been facilitated by the detection of the highly conserved 16S ribosomal RNA present in bacteria. The 16S ribosome is present on bacterial (but not human) cells [15]. Using 16S sequencing it has become apparent that there are a vast number of different bacteria present in the normal human body.The upper respiratory tract (especially the nasopharynx and the mouth) is densely colonized by a large variety of bacteria [16]. The colonization of this microbiome is a dynamic process with frequent turnover of different strains. A number of these bacteria are potentially pathogenic micro-organisms (PPM) if they move into the lower respiratory tract [17]. These potential pathogens may colonize the upper airway for long periods such as *Haemophilus influenzae, Moxarella catarrhalis* and *Streptococcus pneumoniae* or for transient periods such as *Mycoplasma pneumoniae* [18, 19]. A recent study has described that the depletion of the normal microbiome is associated with an increased prevalence of *Corynebacterium tuberculostearium* that mediates rhinosinusitis; this pathogen could potentially have a role in the lower respiratory tract [20].

The lower respiratory tract is directly connected to the upper respiratory tract with frequent micro-aspiration of secretions. With the use of 16S sequencing it has been recently recognized that the lower respiratory tract is in fact not sterile but contains a variety of bacteria [16]. The bacteria present in the lower respiratory tract could be quickly cleared by host defense, may persist as pathogens causing ongoing inflammation or form a lower respiratory tract microbiome (i.e. bacteria exist in symbiosis with the host). It has also become apparent that patients with chronic airways disease (asthma and COPD) have a different spectrum of bacteria in the lower respiratory tract than those with normal lungs [15, 21–25]. These studies have generally found a close correlation between the upper and lower airway microbiome. It should be emphasized that defining the lower airway bacteria is challenging and the studies done so far have had small numbers. The quality of the data varies and studies have used a range of techniques to sample the lower airway. Recent studies of the lower respiratory tract microbiome are listed in Table 1.

**Table 1:** **Studies of the lung microbiome in COPD**

Study	Subjects (n)	Sample	Key findings
Hilty et al. 2010 [21]	8 Control	BAL/Brush	Pathogenic bacteria, e.g. *H. influenzae* in asthma and COPD subjects
	11 Asthma		
	5 COPD		
Charlson et al. 2010 [22]	29 Smoker	Pharynx	Different regions of pharynx have different microbial communities which are changed by smoking
	33 Non-smoker		
Erb-Downward et al. 2011 [23]	9 Control/	BAL	Core pulmonary microbiota in BAL & lung tissue
	5 COPD		
	6 severe COPD		
Sze et al. 2012 [24]	8 Control	Lung tissue	Differences in bacterial communities in the same lung
	8 COPD		
	8 severe COPD		
	8 Cystic fibrosis		
Pragmann et al. 2012 [25]	10 Control	BAL	Detectable bacterial communities in lung tissue that changes in severe COPD
	22 COPD		
Morris et al. 2013 [15]	32 Smoker	Oral wash	Mouth communities different in smoker
	30 Non-smoker		

Several studies have demonstrated the presence of bacteria particularly *H. influenzae* in surgical lung tissue in patients with COPD [26, 27]. The ability of bacteria to invade into the respiratory tract and persist for long periods either between or inside cells may also be potentially important [28].

### The potential role of bacteria in COPD

COPD is characterized by airway inflammation that causes progressive pulmonary damage. The primary risk factor is smoking which has a variety of pathogenic effects on the airway. However it has been recognized that airway inflammation persists after smoking cessation [8]. Hogg et al. have described the presence of lymphoid follicles in the airway walls of ex-smokers that have a key role in driving inflammation and airflow obstruction [9]. These lymphoid follicles are likely to be resulting from the presence of persistent lung antigen but what this could be has not been well defined. Potential antigens include autoimmune antibodies, embedded heavy metals from smoking and bacterial infection [29].

Smoking has a number of long-term effects on the lung host defense that persist after smoking cessation and increase the risk of secondary bacterial infection [30]. These include permanent structural damage and changes in the function of host immune cells including macrophages, NK cells, dendritic cells and lymphocytes.

The ability of bacteria to cause disease is primarily related to overcoming the protective host immune response. Both the structural airway integrity and cellular immune response have a critical role. The airways secrete anti-microbial peptides such as defenses which control microflora [31]. Rhinovirus infections can downregulate the production of these peptides and this may be important in exacerbations of COPD [32].

### Presence of bacteria in exacerbations of COPD

A factor put forward for the relevance of bacteria in COPD is their strong association with exacerbations. Approximately 50% of exacerbations of COPD are associated with the isolation of bacteria from the lower respiratory tract [33, 34]. The dominant bacterium are; *H. influenzae, S. pneumoniae* and *Moxarella catarrhalis*. In advanced disease *P. aeruginosa* becomes more prevalent [34]. There are challenges in obtaining a representative sample; but studies of sputum and bronchoscopy samples using standard culture and molecular techniques have clearly demonstrated that COPD exacerbations are associated with a markedly increased prevalence of bacteria.

The bacteria that are associated with exacerbations could potentially be new strains of bacteria from the upper respiratory tract or from chronic colonization of the lower respiratory tract. Sethi and colleagues have described that the acquisition of a new strain of bacteria (particularly *H. influenzae*) is associated with COPD exacerbations [35, 36]. However only a minority of exacerbations were associated with the acquisition of a new strain. It has been difficult to define, but 38-73% of patients with stable COPD have been described to have the presence of PPMs on baseline sputum [7, 37–41], and on bronchoscopy between 33-50% [42–44]. Exacerbations could arise in the context of these resident bacteria. Similar to the upper airway the lower airway bacterial colonization is a dynamic process with turnover of strains.

A recently described entity is bacterial and viral co-infection. This may be present in up to 25% of exacerbations and is associated with worse clinical outcomes and more severe disease [37, 45, 46]. The viral infection may down-regulate host defense leading to enhanced bacterial proliferation and inflammation. Malia et al. have recently demonstrated that experimental rhinovirus infection is associated with a very high rate of bacterial exacerbation in COPD patients [32].

### Bacteria and decline in lung function

There is indirect evidence correlating the decline in lung function with airway infection and chronic bronchitis in COPD [47, 48]. Chronic bronchitis, respiratory infections and sputum bacterial counts have been shown to be related to decline in lung function in COPD. The studies that have been done in this regard have generally been cross-sectional rather than longitudinal. The demonstration that the clearance of bacteria is associated with improved lung function would be an important advance.

### Role of bacteria in inflammation

COPD is characterized by persistent inflammation that damages lung tissue, causing airflow obstruction. This inflammation persists after smoking. Neutrophils, macrophages and T cells produce mediators such as cytokines, reactive oxygen species and proteases to drive this process [10, 29, 49]. Whilst it is generally accepted that bacteria are very commonly found in the airways in COPD, their role in the inflammatory process is less well defined.

#### In exacerbations of COPD

Two studies have reported increased levels of the inflammatory mediators, interleukin (IL) 8, tumor necrosis factor alpha (TNF-α), neutrophils and elastase with bacteria [50, 51]. Other studies have found that bacterial exacerbations were associated with an increase in a variety of parameters including neutrophils, eosinophils, TNF-α, IL-1, CCL 5, CCL3 and CXCL11 [52–57]. Sethi et al. found that the presence of *H. influenzae* and *M. catarrhalis* was associated with increased sputum inflammatory markers [58].

#### Lower airway bacterial colonization

This may also contribute to chronic airway inflammation. White et al. showed that resolution of inflammation after an exacerbation is dependent on bacterial clearance; when this does not occur there may be ongoing inflammation [59]. Chronic airway colonization has been shown to be related to the frequency and severity of exacerbations [7, 40, 60] and sputum inflammatory markers [41, 60]. In stable patients airway colonization has been demonstrated to have increased levels of airway inflammatory markers including TNF-α, IL-1, IL-6, IL-12, matrix metalloproteinase (MMP), myeloperoxidase and neutrophil elastase [38, 41, 42, 60–62]. The load of bacteria has been described to be related to the exacerbations and inflammation; using both culture [7] and molecular techniques [63] although other researchers have come to different conclusions [36].

Lymphocyte are recognized to have a key role in driving inflammation in COPD but the antigens that activate these cells have not been well defined. We have recently demonstrated that nontypeable *Haemophilus influenzae* (NTHi) strongly activates T cells obtained from surgical lung tissue to cause a proinflammatory, profibrotic response that also enhances airway reactivity [64]. This work suggests that NTHi has a role in driving T cell mediated inflammation in COPD. Factors which suggest a role for bacteria in the pathogenesis of COPD are summarized in listed below.

**Factors that imply a potential role for bacteria in COPD**

1. Present in approximately 50% of lower airway samples in exacerbations
2. Colonize the airways in approximately 33-73% of COPD patients
3. Chronic bronchitis, respiratory infections and sputum bacterial counts are associated with lung function decline
4. Bacteria are associated with increased inflammatory markers in exacerbations
5. Chronic airway colonization is related to the frequency and severity of exacerbations and to levels of airway inflammatory markers
6. Bacteria activate lung T cells in COPD to produce a pro-inflammatory response

### Diagnosis of bacteria and infection

The available literature suggests that bacteria are an important cause of inflammation in COPD however this is probably only in a proportion of patients. COPD is a heterogeneous condition and recent efforts have been made to phenotype this condition. Patients with chronic bronchitis and with frequent exacerbations could represent a phenotype associated with bacterial infection [47, 65]. The evidence that the identification of specific bacteria changes treatment outcomes is minimal. However this may change with better quality studies and newer techniques.

Sputum samples are the first-line investigation used. Stockley et al. have established that the production of purulent sputum that is graded on a color scale (from white to dark green; as a marker of neutrophils) is strongly associated with bacterial infection [66, 67]. A good quality specimen that is representative of the lower airway should have; < 10 squamous epithelial cells and >25 polymorphs in low power field [68, 69]. Sputum culture results have the highest validity when they are consistent with the gram stain (e.g. G-ve bacilli on gram stain accompanied by growth of *P. aeruginosa* on culture) [70, 71]. The use of nucleic acid amplification tests (NAAT) may increase the yield of fastidious pathogens such as *M. pneumoniae* and *C. pneumoniae* [72, 73].

The diagnostic yield may be increased by the use of bronchoscopy. There is a significant issue of potential contamination as the bronchoscope is initially passed through the upper airway. The protected specimen brush is more specific than the bronchoalveolar lavage (BAL) although it does have the issue of only obtaining small specimen amounts [74]. Using a cut off of colony forming units (CFU) of bacteria per ml of specimen (e.g. 10^3^ ml) increases sensitivity [75, 76]. As described previously 16S sequencing has given a new perspective on the lower airway; at this stage it is primarily a research tool but in time is likely to become more mainstream.

There may also be significant merit in taking bronchial biopsies or examining surgical lung specimens for the presence of bacteria as this tissue should be sterile. Patel et al. described the presence of intracellular *H. influenzae* in 14/15 patients having an exacerbation of COPD (and none in controls) [40]. Moller et al. examined the explants of patients with COPD (and other conditions) and found widespread invasion of lung tissue with intracellular *H. influenzae* in half of the subjects [77]. More recently, Droman et al. described the presence of this bacterium in the lung tissue in 40% of patients in all stages of COPD [27].

### Use of antibiotics in COPD

There is a lack of good quality trials concerning the effectiveness of antibiotics for the treatment of COPD and this more than anything has contributed to the controversy about the role of bacteria in the pathogenesis of this condition.

### Use of antibiotics for exacerbations of COPD

There have been a number of problems with the antibiotic trials in COPD exacerbations; particularly with issues of lack of placebo control, poorly defined patient groups, lack of follow-up data and the endpoints measured [78, 79]. In many trials it has been felt that it is not ethical to withhold antibiotics (i.e. use a placebo) so typically designs have compared one antibiotic with another [78, 80].

Perhaps the best study to date is that of Anthonisen et al. who used a randomized, double-blind design [81]. They defined three cardinal symptoms of increasing; 1) dyspnea 2), sputum volume and 3) sputum purulence. Subjects who had two symptoms (type 2) or three symptoms (type 1) had a modest benefit from antibiotics (subjects with one symptom had no benefit).

A recent Cochrane review assessed the use of antibiotics for exacerbations of COPD [11]. Sixteen randomized controlled trials of 2068 subjects were included. In mild exacerbations they found there was evidence of low quality that antibiotics significantly reduced the risk of treatment failure 7 days to one month after treatment initiation. Evidence of high quality demonstrated a significant reduction in treatment failure in in-patients with severe exacerbations. Evidence of moderate quality demonstrated an increased risk of side effects with antibiotics (particularly diarrhea).

A recent trial by Llor et al. evaluated the effect of antibiotics in an ambulatory population of mild-moderate exacerbations [82]. This trial randomized patients to be either treated with amoxicillin/clavulanate (n=117) or placebo (n=91). They found that the use of antibiotics was more effective and significantly prolonged the time to next exacerbation when compared to placebo. This is the first trial to show a clear benefit in the outpatient setting and one of the few trials that has used a placebo.

Other criteria have been proposed as being useful in determining whether antibiotics are indicated for exacerbations. Sputum purulence is correlated with bacterial infection [67] and withholding antibiotics in those without purulent sputum may be safe [67, 83]. Procalcitonin is a marker of bacterial infection and may be a useful guide as to whether antibiotics are indicated [84, 85]. The most commonly used biomarker in COPD is the C-reactive protein (CRP). Llor et al. found that a CRP of > 40 mg/L in the placebo group predicted clinical failure [82]. The role of biomarkers as a marker of inflammation in COPD is an area of active research with several recent large studies published. MacNee has recently reviewed this topic [86] and has highlighted the role of the lung-specific factors CCL-18/PARC and SP-D, which are closely correlated with clinical outcomes (e.g. exacerbations) and anti-inflammatory therapy; fibrinogen whilst less organ specific may also be helpful.

### Use of antibiotics in stable COPD

There is increasing evidence that bacteria contributes to lung inflammation both in exacerbations and in stable COPD. Antibiotics have used for the treatment of chronic bronchitis for many years with trials first done 50 years. Staykova et al. reviewed the early trials and found a small reduction in days of illness in exacerbations [87]. Due to lack of good data and concerns about antibiotic resistance this approach was rarely used for the management of stable COPD. However this has changed recently with a series of trials that have used macrolides.

Seemungal et al. assessed a cohort of 109 patients randomized to treatment with erythromycin or placebo for a year and found there was a significant reduction in exacerbations in the erythromycin group [88]. A major study of 1142 subjects who were randomized to treatment with azithromycin 250mg daily or placebo for a year found a significant reduction in exacerbations and improvement in quality of life in the treatment arm [12]. Several other studies have found that macrolides reduce exacerbations of COPD [89–91]. The mechanisms by which macrolides reduce exacerbations are unknown.

There is significant overlap with bronchiectasis and COPD. Two trials have reported in COPD a prevalence of bronchiectasis of 29% and 50% [92, 93]. Two studies have found improved outcomes in cohorts of bronchiectasis patients treated with macrolides. Wong et al. demonstrated that the use of azithromycin at 500mg three times a week significantly reduced exacerbations when compared to control [94]. Similarly Serisier et al. found that the use of erythromycin 400mg twice daily for 12 months reduced exacerbations when compared to control [95].

There are some potential problems with the use of macrolides to prevent exacerbations [96]. Azithromycin has a mildly increased risk of hearing deficits and prolonged QT syndrome with a one study reported a slightly increased risk of cardiac death [97]. However a more recent study found no increased risk of cardiac death [98]. Macrolides should probably be given with caution to subjects with significant cardiac or vascular disease. There are drug interactions especially with statins, warfarin and amiodarone. There is also an increased risk of antibiotic resistance including to *S. pneumoniae* and *Mycobacterium avium* complex. Wenzel et al. recommend that macrolides (azithromycin) should be considered as maintenance therapy in subjects who have at least 2 exacerbations in past year despite optimal therapy and no significant contraindications [96].

### Other therapies to treat infection in COPD

Antibiotics are a limited resource and should be used when there is a strong clinical indication. There is a significant need for other therapeutic modalities to treat infection in the context of COPD.

### Vaccination for COPD

The influenza and pneumococcal vaccines are a frequent part of standard management of COPD [99]. Viral influenza is associated with increased exacerbations of COPD and a higher risk of hospitalization. A Cochrane review in 2006 found that vaccination resulted in a mildly decreased risk of exacerbations. The vaccine may be given as inactive or attenuated forms and both forms appear safe with no clear differences in efficacy [100].

*Streptococcus pneumoniae* is a major cause of acute exacerbations of COPD and may also colonize the lower airway. The vaccine has two forms, the valent polysaccharide vaccine and the newer conjugated vaccine. The conjugated vacccine induces a stronger immune response and may potentially be more effective. Three retrospective studies demonstrated a reduction in the incidence of pneumonia, hospitalizations and invasive disease [101–103]. A Cochrane review found a reduction in invasive pulmonary disease after pneumococcal vaccination but no reduction in all-cause pneumonia or mortality [104].

In addition to influenza and pneumococcal vaccination the Centers for Disease Control (CDC) and American Society for Infectious Diseases (ASID) also recommend Pertussis and Herpes Zoster vaccination in patients with COPD. The evidence supporting the use of Pertussis and Herpes Zoster vaccination in this context is minimal.

The nontypeable form of *Haemophilus influenzae* is the dominant bacterium in patients with COPD and there have been studies of the use of vaccination against this pathogen intermittently for a number of years. An inactivated vaccine has been previously shown to reduce the incidence of acute exacerbations in COPD but was not widely used [105]. A more recent study has again demonstrated that oral vaccination is safe and reduces severe exacerbations [106]. A successful vaccine against *H. influenzae* would be a major advance in management.

### Use of corticosteroids

Systemic corticosteroids are standard therapy for the treatment of COPD exacerbations. The combination of corticosteroids and antibiotics for exacerbations has been demonstrated to improve outcomes when compared to either agent alone [107].

The long-term use of corticosteroids on infection in COPD is not clear-cut. Corticosteroids are potentially beneficial by reducing inflammation, however this results in a down-regulation of host defense and increased risk of infection. Maintenance therapy with inhalational corticosteroids has been shown to reduce the number of exacerbations but conversely increase the rate of pneumonia [108].

### Other agents

As the viral-bacterial co-infection is associated with worse outcomes in COPD there may be a role for antivirals in the treatment of exacerbations. Neuraminidase-inhibitors for the treatment of influenza may be beneficial although there appears to be no clear literature to guide this choice. Recently drugs have been developed against the rhinovirus including the capsid-binding agent, pleconaril [109].

As bacterial infection produces inflammation, the use of anti-inflammatory agents may be useful. Phosphodiesterase inhibitors such as roflumilast and cilomilast reduce inflammation and exacerbations in COPD [110, 111]. Antioxidants such as N-acetylcysteine in subgroup analysis may reduce exacerbations [112]. Whether these effects are relevant to bacterial infection remains to be determined.

The intestinal microbiome has recently been recognized to have general effects on host immunity including in the lung. Therefore the use of probiotics to enhance normal intestinal flora may have potentially beneficial effects on lung inflammation. Forsythe has reviewed this topic; whilst animal data has shown promising results for the use of probiotics to reduce lung inflammation, human studies have so far been inconclusive [113].

## Conclusions

Increasing evidence has demonstrated that bacteria are very common in the lower respiratory tract of patients with COPD and cause inflammation. Antibiotics have a clear role in the treatment of a proportion of subjects. There are still very large gaps in understanding how bacteria contribute to inflammation in COPD and what are the best methods to diagnose their presence in individual patients. Antibiotics are a limited resource and establishing how they should best be utilized to treat infection in COPD will be a major challenge. The development of alternative therapies is likely to be critical to long-term improvement in management.
